# MicroRNA guardians of the posterior cingulate cortex delay cognitive deterioration in elders

**DOI:** 10.1093/braincomms/fcae099

**Published:** 2024-03-25

**Authors:** Hermona Soreq

**Affiliations:** The Edmond & Lily Safra Center for Brain Sciences, The Hebrew University of Jerusalem, Jerusalem 9190401, Israel; Alexander Silberman Institute of Life Sciences, The Hebrew University of Jerusalem, Jerusalem 9190401, Israel

## Abstract

This scientific commentary refers to ‘MicroRNA profiles of pathology and resilience in posterior cingulate cortex of cognitively intact elders’, by Kelley *et al*. (https://doi.org/10.1093/braincomms/fcae082).

**This scientific commentary refers to ‘MicroRNA profiles of pathology and resilience in posterior cingulate cortex of cognitively intact elders’, by Kelley *et al*. (https://doi.org/10.1093/braincomms/fcae082).**

The posterior cingulate cortex (PCC) holds personal memories, whose retrieval fails early in the progression of Alzheimer’s disease.^1,2^ In their recent impressive article in *Brain Communications*, Kelley *et al*.^3^ challenge an often-raised question: how come certain elderly individuals maintain impeccable personal memories in spite of presenting measurable brain pathology following the Braak scale that is characteristic of the early phase of Alzheimer’s disease? To address this topic, Kelley *et al*. analysed both long mRNA transcriptomics and short non-coding RNA-sequencing data from post-mortem PCC tissues of patients with or without reported functioning memories.

In selecting the PCC as their primary brain region of interest and RNA-sequencing datasets as their research approach, Kelley and co-authors challenged the working hypothesis that both protein-coding RNAs and non-coding RNA regulators, or co-altered regulation thereof, may offer a potential explanation to this surprising phenomenon; and indeed, their findings identified conspicuously distinct transcriptomic profiles for PCC mRNAs from patient groups with and without decline of personal memories, highlighting altered levels of synaptic and ATP-related mRNA transcripts as well as microRNA regulators thereof in the PCC from patients with advanced Braak profiles with or without maintenance of their past personal memories.

Can early Alzheimer’s disease patients hyper-activate their brain neurons to maintain personal memories even though their Braak pathology has progressed? An earlier and less discriminative whole brain approach to this query referred to the difference between pathology-presenting Alzheimer’s disease patients with and without cognitive failure. That was done by analysing their neuronal expressed transcripts and profiling the lipid composition in their brains; in that study, hyper-activated neuronal gene expression and co-altered brain lipid distributions emerged as the discriminative elements, compatible with related publications by others.^4^ The indication was hence that cortical neurons in some patients, for yet unknown reasons, may selectively become hyperactive, which could assist the maintenance of personal memories and delay cognitive deterioration yet involves altered lipid composition in the affected brain.

Yet more recently, a study combining long and small RNA-sequencing data of the religious order patient brains was focused on short RNA contents of the cholinergic nucleus accumbens derived from male and female Alzheimer’s disease patients as related to their previously assessed cognitive levels. Three points of special interest emerged in that study: first and foremost, the features of both microRNAs (miRs) and the lately re-discovered transfer RNA fragments (tRFs), part of which may target cholinergic mRNA transcripts, emerged as being differently regulated in these intriguing groups of patients; second, the analysis identified the great majority of tRFs targeting cholinergic transcripts as originated from mitochondrial tRNA genes. And third, women but not men living with Alzheimer’s disease emerged as presenting declined levels of cholinergic transcripts-targeted tRFs, but not microRNAs in their nucleus accumbens. Importantly, this loss was related specifically to the cognitive decline of those women, which has been known for some time to escalate more rapidly in women than men.^5^

Taken together, both Kelley *et al*.’s article^3^ and the above cited studies^4,5^ endorse a working hypothesis stating that both global alterations in the microRNA profiles in the PCC and loss of mitochondrially-originated tRFs targeted to cholinergic mRNA transcripts in the nucleus accumbens present functional links with cognition, and in the current study, particularly with one’s personal memories; furthermore, those studies connected both the early loss of personal memories in Alzheimer’s disease patients and the rapid cognitive decline of women living with Alzheimer’s disease with small regulatory RNA activities. Supporting this notion, recent studies by Tzur *et al*.^6^ showed co-altered levels of microRNAs and tRFs in response to both cholinergic and metabolic stresses in general.

The cholinergic elements in this complex picture raise two more important points: first, the reservoir of mitochondrially-transcribed tRNAs may be depleted with age in some, but not all aged individuals; therefore, brain tRFs levels may undergo individual aging-related rates of decline^7^ and their protective effects over the cholinergic signalling process may be depleted with age. Second, several recent studies over the past few years demonstrate increased susceptibility to cognitive decline in aged patients treated with anti-cholinergic therapeutics.^8^ Taken together, these studies call for special precautions. First, administering anti-cholinergic medications to aged individuals may sometimes, but not always, improve their prospects. And second, when Alzheimer’s disease commences, some patients may activate personal memories-preserving processes and delay the cognitive damage accompanying disease progression, albeit for a yet undefined time.

Last, but not least, this study highlights the importance of small non-coding RNAs, including both microRNAs and tRFs to orchestrate the maintenance of our brain’s activities and associate them with delayed onset of the cognitive disease symptoms. Notably, our genome includes many thousands of primate-specific microRNA genes, whose transcript products interact with complementary sequence regions in target messenger RNAs and block their translation (Barbash *et al*.,^9^ Fig. 1). Importantly, both our nuclear and mitochondrial genomes possess numerous microRNA and tRNA genes of their own, and tRNAs break down to yield tRFs. Therefore, the current and previous studies jointly indicate that impaired mitochondrial genes in particular may reflect susceptibility to early onset of personal memory losses and/or rapid cognitive decline in aged individual patients, mainly women living with Alzheimer’s disease. In conclusion, Kelley *et al*.’s article deserves special attention.

**Figure 1:**
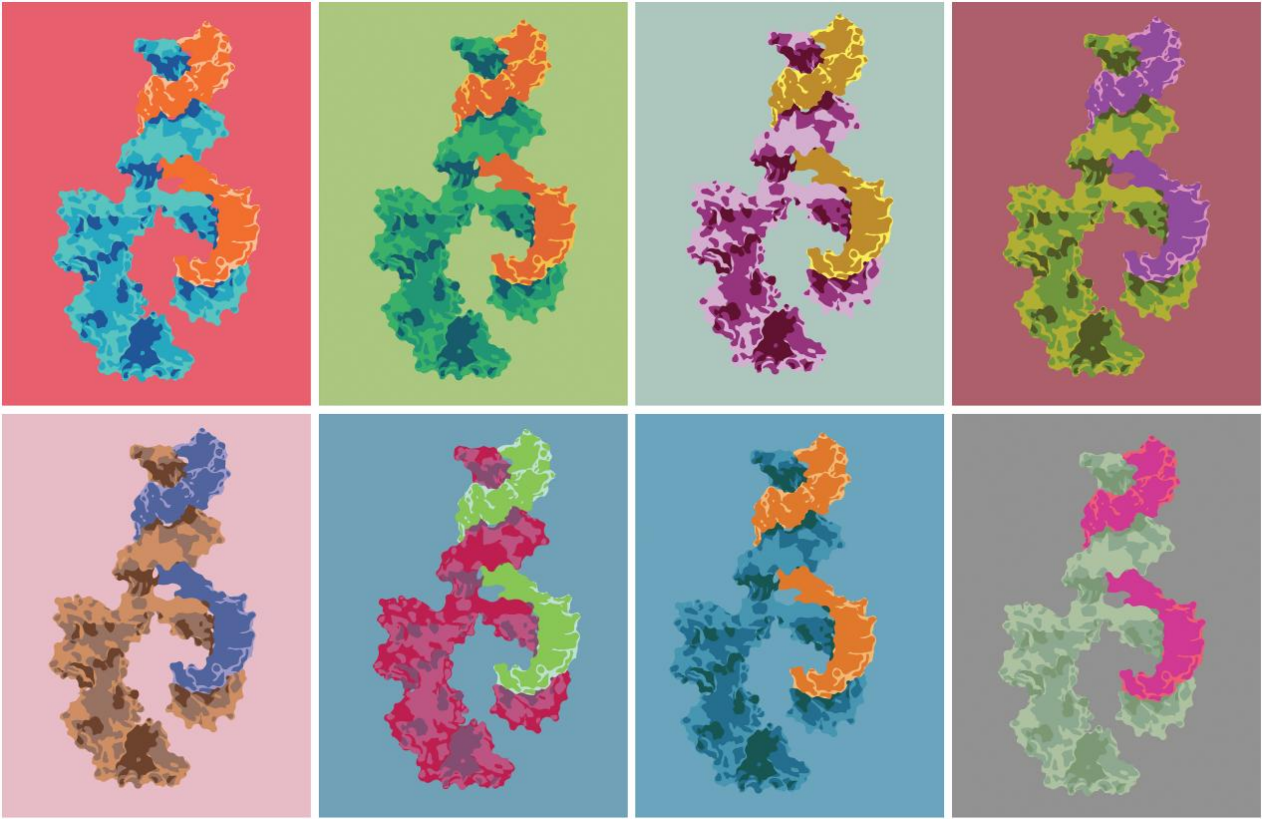
**Small regulatory RNAs may be causally involved.** Shown is a graphical representation of the conceptual activities of small non-coding regulatory RNAs. Many such RNAs, both microRNAs and tRFs emerge as contributing to the loss of personal cognitive memories in the brain of some, but not all, Alzheimer’s disease patients, reflecting age-related decline due to failure to interact with their coding mRNA targets and/or altering the brain’s lipid composition.

## Data Availability

There are no new data associated with this article.
